# 3-[(1-Isobutyl-1*H*-imidazo[4,5-*c*]quinolin-4-yl)amino]­benzoic acid

**DOI:** 10.1107/S1600536811028765

**Published:** 2011-07-30

**Authors:** Hoong-Kun Fun, Tara Shahani, Dinesh, Reshma Kayarmar, G. K. Nagaraja

**Affiliations:** a X-ray Crystallography Unit, School of Physics, Universiti Sains Malaysia, 11800 USM, Penang, Malaysia; b Department of Chemistry, Mangalore University, Karnataka, India

## Abstract

In the title compound, C_21_H_20_N_4_O_2_, the statistically planar 1*H*-limidazole ring [maximum deviation = 0.003 (1) Å] makes dihedral angles of 1.33 (9) and 8.23 (7)°, respectively, with the essentially planar fused pyridine ring [maximum devation = 0.018 (1) Å] and the pendant benzene ring, which is attached to the pyridine ring by an —NH— group. An intra­molecular C—H⋯N inter­action, which generates an S(6) ring, helps to estalish the mol­ecular conformation. In the crystal, the mol­ecules are linked by N—H⋯O, C—H⋯O and O—H—N hydrogen bonds, which generate bifurcated *R* ^1^_2_(6) and *R* ^2^_2_(9) ring motifs, resulting in supra­molecular [001] chains. The crystal structure also features weak π–π stacking [centroid–centroid distance = 3.5943 (9) Å] and C—H⋯π inter­actions.

## Related literature

For our previous study of a related structure and background references, see: Loh *et al.* (2011 ▶). For a further related structure, see: Rasmussen *et al.* (2009 ▶). For hydrogen-bond motifs, see: Bernstein *et al.* (1995 ▶). For reference bond-length data, see: Allen *et al.* (1987 ▶).
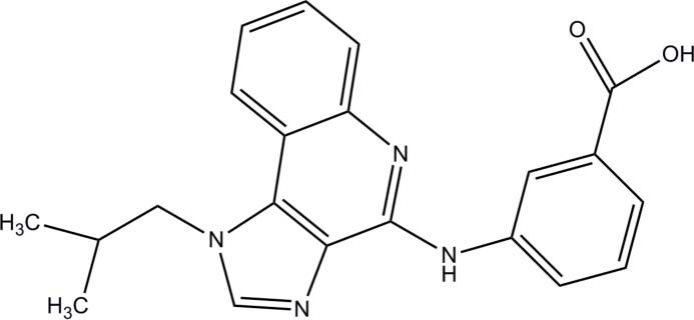


## Experimental

### 

#### Crystal data

C_21_H_20_N_4_O_2_
*M* *_r_* = 360.41
Monoclinic, 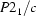

*a* = 9.6440 (1) Å
*b* = 15.1496 (2) Å
*c* = 14.5286 (2) Å
β = 123.927 (1)°
*V* = 1761.28 (4) Å^3^
*Z* = 4
Mo *K*α radiation
μ = 0.09 mm^−1^
*T* = 296 K
0.41 × 0.26 × 0.22 mm

#### Data collection

Bruker APEXII DUO CCD diffractometer
Absorption correction: multi-scan (*SADABS*; Bruker, 2009 ▶) *T*_min_ = 0.964, *T*_max_ = 0.980
23777 measured reflections
5859 independent reflections
4504 reflections with *I* > 2σ(*I*)
*R*_int_ = 0.035

#### Refinement

*R*[*F* ^2^ > 2σ(*F* ^2^)] = 0.051
*wR*(*F* ^2^) = 0.140
*S* = 1.04
5859 reflections
254 parameters
H atoms treated by a mixture of independent and constrained refinement
Δρ_max_ = 0.51 e Å^−3^
Δρ_min_ = −0.22 e Å^−3^

### 

Data collection: *APEX2* (Bruker, 2009 ▶); cell refinement: *SAINT* (Bruker, 2009 ▶); data reduction: *SAINT*; program(s) used to solve structure: *SHELXTL* (Sheldrick, 2008 ▶); program(s) used to refine structure: *SHELXTL*; molecular graphics: *SHELXTL*; software used to prepare material for publication: *SHELXTL* and *PLATON* (Spek, 2009) ▶.

## Supplementary Material

Crystal structure: contains datablock(s) global, I. DOI: 10.1107/S1600536811028765/hb5954sup1.cif

Structure factors: contains datablock(s) I. DOI: 10.1107/S1600536811028765/hb5954Isup2.hkl

Supplementary material file. DOI: 10.1107/S1600536811028765/hb5954Isup3.cml

Additional supplementary materials:  crystallographic information; 3D view; checkCIF report

## Figures and Tables

**Table 1:** Hydrogen-bond geometry (Å, °)

*D*—H⋯*A*	*D*—H	H⋯*A*	*D*⋯*A*	*D*—H⋯*A*
N1—H1*N*1⋯O1^i^	0.860 (16)	2.114 (17)	2.9436 (14)	161.8 (15)
O2—H1*O*2⋯N4^ii^	0.99 (2)	1.71 (2)	2.6926 (13)	172 (2)
C3—H3*A*⋯O1^i^	0.93	2.42	3.1998 (15)	142
C5—H5*A*⋯N2	0.93	2.21	2.8337 (16)	123
C1—H1*A*⋯*Cg*2^iii^	0.93	2.94	3.3877 (14)	111

Symmetry codes: (i) 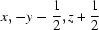; (ii) 
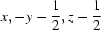
; (iii) 
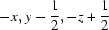
.

**Fig. 1.**
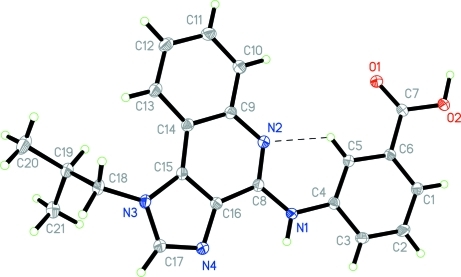
The molecular structure of the title compound, showing 30% probability displacement ellipsoids. The intramolecular hydrogen bond is shown by a dashed line.

**Fig. 2.**
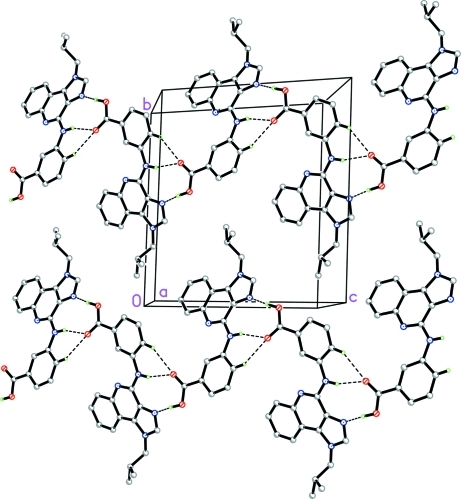
The crystal packing of the title compound, viewed approximately along a axis. Intermolecular hydrogen bonds link the molecules into chains along [001].

## supplementary crystallographic information

### Comment

As part of our ongoing studies of imidazoquinoline derivatives with possible pharmacological activity (Loh *et al.*, 2011), we now report the synthesis and structure of the title compound, (I).

The asymmetric unit of the title compound is shown in Fig. 1. The essentially planar 1*H*-limidazole ring (N3/N4/C15–C17) [maximum deviation = 0.003 (1) Å for atom C16] makes dihedral angles of 1.33 (9)° and 8.23 (7)° respectively with the essentially planar pyridine ring (N2/C8/C9/C14–C16 [maximum devation = 0.018 (1) Å for atom C8] and benzene ring (C1–C6) which is attached to the pridine ring by dimethylamine group (N1/C4/C8). The bond lengths (Allen *et al.*, 1987) and angles are within normal ranges and is comparable to a closely the related structure (Rasmussen *et al.*, 2009).

In the crystal structure (Fig. 2), the molecules are linked by intermolecular N1—H1N1···O1, C3—H3A···O1 and C5—H5A···N2 hydrogen bonds generating bifurcated *R*^1^_2_(6) and *R*^2^_2_(9) ring motifs, resulting in supramolecular [001] chains. Furthermore, the crystal structure is stabilized by weak π···π interactions between 1*H*-limidazole andpyridine rings [centroid–centroid distance = 3.5943 (9) Å; 1 - *x*, -*y*, 1 - *z*] and C—H···π interactions, involving *Cg*1 (N3/N4/C15–C17) and *Cg*2 (C9–C14) rings.

### Experimental

To a solution of 4-chloro-1-(2-metylpropyl)-1*H*-imidazo [4,5-*c*] quinoline (0.1 mol) in ethanol (30 ml), 3-Aminobenzoic acid (0.1 mol) was added and the reaction mixture was refluxed for 24 h. It was then concentrated, cooled and poured over crushed ice to get the precipitate which was filtered, washed with water and recrystallized from ethanol to yield colourless blocks of (I). *Mp*: 485–487 K.

### Refinement

Atoms H1N1 and H1O2 were located from a difference Fourier maps and refined freely [N–H = 0.860 (16) and O–H = 0.99 (2) Å]. The remaining H atoms were positioned geometrically [C–H = 0.93–0.97 Å] and were refined using a riding model, with *U*_iso_(H) = 1.2 or 1.5 *U*_eq_(C). A rotating group model was used for the methyl group.

### Crystal data

**Table:**

C_21_H_20_N_4_O_2_	*F*(000) = 760
*M**_r_* = 360.41	*D*_x_ = 1.359 Mg m^−^^3^
Monoclinic, *P*2_1_/*c*	Mo *K*α radiation, λ = 0.71073 Å
Hall symbol: -P 2ybc	Cell parameters from 5489 reflections
*a* = 9.6440 (1) Å	θ = 2.2–32.1°
*b* = 15.1496 (2) Å	µ = 0.09 mm^−^^1^
*c* = 14.5286 (2) Å	*T* = 296 K
β = 123.927 (1)°	Block, colourless
*V* = 1761.28 (4) Å^3^	0.41 × 0.26 × 0.22 mm
*Z* = 4	

### Data collection

**Table:**

Bruker APEXII DUO CCD diffractometer	5859 independent reflections
Radiation source: fine-focus sealed tube	4504 reflections with *I* > 2σ(*I*)
graphite	*R*_int_ = 0.035
φ and ω scans	θ_max_ = 31.5°, θ_min_ = 2.2°
Absorption correction: multi-scan (*SADABS*; Bruker, 2009)	*h* = −13→14
*T*_min_ = 0.964, *T*_max_ = 0.980	*k* = −20→22
23777 measured reflections	*l* = −21→18

### Refinement

**Table:**

Refinement on *F*^2^	Primary atom site location: structure-invariant direct methods
Least-squares matrix: full	Secondary atom site location: difference Fourier map
*R*[*F*^2^ > 2σ(*F*^2^)] = 0.051	Hydrogen site location: inferred from neighbouring sites
*wR*(*F*^2^) = 0.140	H atoms treated by a mixture of independent and constrained refinement
*S* = 1.04	*w* = 1/[σ^2^(*F*_o_^2^) + (0.0764*P*)^2^ + 0.3392*P*] where *P* = (*F*_o_^2^ + 2*F*_c_^2^)/3
5859 reflections	(Δ/σ)_max_ < 0.001
254 parameters	Δρ_max_ = 0.51 e Å^−^^3^
0 restraints	Δρ_min_ = −0.22 e Å^−^^3^

### Special details

**Table:**

Geometry. All e.s.d.'s (except the e.s.d. in the dihedral angle between two l.s. planes) are estimated using the full covariance matrix. The cell e.s.d.'s are taken into account individually in the estimation of e.s.d.'s in distances, angles and torsion angles; correlations between e.s.d.'s in cell parameters are only used when they are defined by crystal symmetry. An approximate (isotropic) treatment of cell e.s.d.'s is used for estimating e.s.d.'s involving l.s. planes.
Refinement. Refinement of *F*^2^ against ALL reflections. The weighted *R*-factor *wR* and goodness of fit *S* are based on *F*^2^, conventional *R*-factors *R* are based on *F*, with *F* set to zero for negative *F*^2^. The threshold expression of *F*^2^ > σ(*F*^2^) is used only for calculating *R*-factors(gt) *etc*. and is not relevant to the choice of reflections for refinement. *R*-factors based on *F*^2^ are statistically about twice as large as those based on *F*, and *R*- factors based on ALL data will be even larger.

### Fractional atomic coordinates and isotropic or equivalent isotropic displacement parameters (Å^2^)

**Table:**

	*x*	*y*	*z*	*U*_iso_*/*U*_eq_	
O1	0.14729 (12)	−0.33971 (6)	0.12947 (8)	0.0215 (2)	
O2	0.15417 (11)	−0.47845 (6)	0.18402 (7)	0.01792 (19)	
N1	0.16268 (13)	−0.15414 (6)	0.43284 (9)	0.0156 (2)	
N2	0.24570 (13)	−0.09564 (6)	0.32204 (8)	0.0155 (2)	
N3	0.33813 (12)	0.13649 (6)	0.49615 (8)	0.0148 (2)	
N4	0.22805 (13)	0.02550 (7)	0.53736 (8)	0.0153 (2)	
C1	0.07884 (15)	−0.42262 (8)	0.33507 (10)	0.0170 (2)	
H1A	0.0624	−0.4821	0.3160	0.020*	
C2	0.05934 (17)	−0.39045 (8)	0.41686 (11)	0.0206 (3)	
H2A	0.0269	−0.4287	0.4516	0.025*	
C3	0.08756 (16)	−0.30209 (8)	0.44747 (10)	0.0188 (2)	
H3A	0.0752	−0.2822	0.5030	0.023*	
C4	0.13454 (14)	−0.24239 (7)	0.39563 (10)	0.0142 (2)	
C5	0.15066 (14)	−0.27426 (8)	0.31152 (9)	0.0142 (2)	
H5A	0.1794	−0.2358	0.2748	0.017*	
C6	0.12366 (14)	−0.36354 (7)	0.28254 (9)	0.0136 (2)	
C7	0.14233 (14)	−0.39209 (8)	0.19153 (9)	0.0144 (2)	
C8	0.21835 (14)	−0.08493 (7)	0.40042 (9)	0.0141 (2)	
C9	0.30995 (14)	−0.02752 (8)	0.29402 (10)	0.0149 (2)	
C10	0.33503 (16)	−0.04493 (8)	0.20876 (10)	0.0190 (2)	
H10A	0.3056	−0.0997	0.1740	0.023*	
C11	0.40222 (16)	0.01784 (8)	0.17665 (11)	0.0207 (3)	
H11A	0.4170	0.0056	0.1199	0.025*	
C12	0.44865 (16)	0.10040 (8)	0.22899 (11)	0.0198 (2)	
H12A	0.4963	0.1422	0.2078	0.024*	
C13	0.42430 (15)	0.12010 (8)	0.31138 (10)	0.0173 (2)	
H13A	0.4547	0.1753	0.3451	0.021*	
C14	0.35331 (14)	0.05705 (8)	0.34531 (9)	0.0143 (2)	
C15	0.31713 (14)	0.06692 (7)	0.42760 (9)	0.0135 (2)	
C16	0.24962 (14)	−0.00111 (8)	0.45501 (9)	0.0139 (2)	
C17	0.28305 (15)	0.10753 (8)	0.55894 (10)	0.0163 (2)	
H17A	0.2841	0.1423	0.6121	0.020*	
C18	0.41403 (15)	0.22270 (8)	0.50622 (10)	0.0168 (2)	
H18A	0.5132	0.2142	0.5056	0.020*	
H18B	0.4490	0.2484	0.5772	0.020*	
C19	0.29783 (15)	0.28790 (8)	0.41406 (10)	0.0175 (2)	
H19A	0.2461	0.2576	0.3425	0.021*	
C20	0.40172 (18)	0.36485 (9)	0.41590 (13)	0.0277 (3)	
H20A	0.4829	0.3431	0.4029	0.041*	
H20B	0.4579	0.3934	0.4869	0.041*	
H20C	0.3297	0.4064	0.3590	0.041*	
C21	0.15965 (15)	0.31946 (8)	0.42709 (11)	0.0211 (3)	
H21A	0.0850	0.3575	0.3663	0.032*	
H21B	0.2080	0.3513	0.4956	0.032*	
H21C	0.0989	0.2695	0.4276	0.032*	
H1N1	0.1529 (19)	−0.1435 (11)	0.4871 (14)	0.021 (4)*	
H1O2	0.171 (3)	−0.4938 (15)	0.1246 (18)	0.052 (6)*	

### Atomic displacement parameters (Å^2^)

**Table:**

	*U*^11^	*U*^22^	*U*^33^	*U*^12^	*U*^13^	*U*^23^
O1	0.0341 (5)	0.0166 (4)	0.0218 (5)	0.0039 (4)	0.0207 (4)	0.0028 (3)
O2	0.0250 (4)	0.0134 (4)	0.0200 (4)	0.0004 (3)	0.0155 (4)	−0.0017 (3)
N1	0.0232 (5)	0.0119 (4)	0.0170 (5)	−0.0023 (4)	0.0145 (4)	−0.0020 (3)
N2	0.0192 (5)	0.0131 (5)	0.0163 (5)	0.0004 (4)	0.0112 (4)	0.0007 (3)
N3	0.0184 (5)	0.0110 (4)	0.0150 (5)	−0.0013 (3)	0.0094 (4)	−0.0002 (3)
N4	0.0184 (5)	0.0141 (5)	0.0147 (4)	−0.0001 (3)	0.0100 (4)	0.0003 (3)
C1	0.0215 (5)	0.0135 (5)	0.0182 (5)	−0.0030 (4)	0.0125 (5)	−0.0018 (4)
C2	0.0298 (6)	0.0166 (6)	0.0220 (6)	−0.0062 (5)	0.0186 (5)	−0.0018 (4)
C3	0.0265 (6)	0.0170 (6)	0.0200 (6)	−0.0043 (4)	0.0174 (5)	−0.0031 (4)
C4	0.0149 (5)	0.0123 (5)	0.0148 (5)	−0.0012 (4)	0.0079 (4)	−0.0009 (4)
C5	0.0162 (5)	0.0125 (5)	0.0151 (5)	−0.0001 (4)	0.0094 (4)	0.0002 (4)
C6	0.0145 (5)	0.0135 (5)	0.0130 (5)	0.0005 (4)	0.0077 (4)	−0.0002 (4)
C7	0.0136 (5)	0.0143 (5)	0.0146 (5)	0.0008 (4)	0.0074 (4)	−0.0017 (4)
C8	0.0153 (5)	0.0119 (5)	0.0144 (5)	−0.0002 (4)	0.0078 (4)	0.0006 (4)
C9	0.0160 (5)	0.0141 (5)	0.0149 (5)	0.0015 (4)	0.0087 (4)	0.0020 (4)
C10	0.0236 (6)	0.0170 (6)	0.0196 (6)	0.0031 (4)	0.0141 (5)	0.0015 (4)
C11	0.0257 (6)	0.0214 (6)	0.0198 (6)	0.0050 (5)	0.0158 (5)	0.0049 (5)
C12	0.0207 (6)	0.0195 (6)	0.0226 (6)	0.0030 (4)	0.0143 (5)	0.0073 (5)
C13	0.0177 (5)	0.0153 (5)	0.0192 (6)	0.0008 (4)	0.0106 (5)	0.0033 (4)
C14	0.0140 (5)	0.0142 (5)	0.0142 (5)	0.0014 (4)	0.0077 (4)	0.0023 (4)
C15	0.0145 (5)	0.0112 (5)	0.0140 (5)	0.0004 (4)	0.0075 (4)	0.0003 (4)
C16	0.0155 (5)	0.0131 (5)	0.0134 (5)	0.0008 (4)	0.0082 (4)	0.0006 (4)
C17	0.0209 (5)	0.0138 (5)	0.0155 (5)	−0.0009 (4)	0.0111 (5)	−0.0005 (4)
C18	0.0182 (5)	0.0119 (5)	0.0189 (6)	−0.0029 (4)	0.0094 (5)	−0.0003 (4)
C19	0.0194 (5)	0.0134 (5)	0.0197 (6)	0.0002 (4)	0.0109 (5)	0.0022 (4)
C20	0.0284 (7)	0.0178 (6)	0.0411 (8)	0.0009 (5)	0.0220 (6)	0.0083 (5)
C21	0.0194 (6)	0.0175 (6)	0.0245 (6)	−0.0001 (4)	0.0111 (5)	−0.0006 (5)

### Geometric parameters (Å, °)

**Table:**

O1—C7	1.2219 (15)	C9—C10	1.4131 (17)
O2—C7	1.3231 (14)	C9—C14	1.4225 (16)
O2—H1O2	0.99 (2)	C10—C11	1.3709 (18)
N1—C8	1.3755 (15)	C10—H10A	0.9300
N1—C4	1.4106 (15)	C11—C12	1.4012 (18)
N1—H1N1	0.860 (16)	C11—H11A	0.9300
N2—C8	1.3145 (15)	C12—C13	1.3750 (18)
N2—C9	1.3771 (15)	C12—H12A	0.9300
N3—C17	1.3592 (15)	C13—C14	1.4146 (16)
N3—C15	1.3852 (14)	C13—H13A	0.9300
N3—C18	1.4641 (15)	C14—C15	1.4280 (17)
N4—C17	1.3185 (15)	C15—C16	1.3907 (16)
N4—C16	1.3838 (15)	C17—H17A	0.9300
C1—C2	1.3908 (17)	C18—C19	1.5327 (16)
C1—C6	1.3923 (16)	C18—H18A	0.9700
C1—H1A	0.9300	C18—H18B	0.9700
C2—C3	1.3887 (17)	C19—C21	1.5238 (18)
C2—H2A	0.9300	C19—C20	1.5276 (18)
C3—C4	1.4041 (16)	C19—H19A	0.9800
C3—H3A	0.9300	C20—H20A	0.9600
C4—C5	1.4008 (16)	C20—H20B	0.9600
C5—C6	1.3973 (16)	C20—H20C	0.9600
C5—H5A	0.9300	C21—H21A	0.9600
C6—C7	1.4945 (16)	C21—H21B	0.9600
C8—C16	1.4371 (16)	C21—H21C	0.9600
			
C7—O2—H1O2	111.6 (13)	C13—C12—C11	120.54 (11)
C8—N1—C4	128.29 (10)	C13—C12—H12A	119.7
C8—N1—H1N1	115.8 (11)	C11—C12—H12A	119.7
C4—N1—H1N1	115.7 (11)	C12—C13—C14	120.52 (11)
C8—N2—C9	120.30 (10)	C12—C13—H13A	119.7
C17—N3—C15	106.38 (10)	C14—C13—H13A	119.7
C17—N3—C18	125.57 (10)	C13—C14—C9	118.92 (11)
C15—N3—C18	127.95 (10)	C13—C14—C15	127.81 (11)
C17—N4—C16	104.21 (10)	C9—C14—C15	113.27 (10)
C2—C1—C6	118.33 (11)	N3—C15—C16	105.18 (10)
C2—C1—H1A	120.8	N3—C15—C14	132.51 (11)
C6—C1—H1A	120.8	C16—C15—C14	122.30 (10)
C3—C2—C1	121.03 (11)	N4—C16—C15	110.61 (10)
C3—C2—H2A	119.5	N4—C16—C8	130.39 (11)
C1—C2—H2A	119.5	C15—C16—C8	118.97 (11)
C2—C3—C4	120.89 (11)	N4—C17—N3	113.62 (11)
C2—C3—H3A	119.6	N4—C17—H17A	123.2
C4—C3—H3A	119.6	N3—C17—H17A	123.2
C5—C4—C3	118.17 (11)	N3—C18—C19	113.99 (10)
C5—C4—N1	124.73 (10)	N3—C18—H18A	108.8
C3—C4—N1	117.10 (11)	C19—C18—H18A	108.8
C6—C5—C4	120.25 (11)	N3—C18—H18B	108.8
C6—C5—H5A	119.9	C19—C18—H18B	108.8
C4—C5—H5A	119.9	H18A—C18—H18B	107.6
C1—C6—C5	121.31 (11)	C21—C19—C20	111.64 (11)
C1—C6—C7	121.77 (10)	C21—C19—C18	110.94 (10)
C5—C6—C7	116.91 (10)	C20—C19—C18	108.90 (10)
O1—C7—O2	122.72 (11)	C21—C19—H19A	108.4
O1—C7—C6	122.59 (10)	C20—C19—H19A	108.4
O2—C7—C6	114.69 (10)	C18—C19—H19A	108.4
N2—C8—N1	120.53 (10)	C19—C20—H20A	109.5
N2—C8—C16	120.36 (10)	C19—C20—H20B	109.5
N1—C8—C16	119.10 (10)	H20A—C20—H20B	109.5
N2—C9—C10	116.40 (11)	C19—C20—H20C	109.5
N2—C9—C14	124.70 (11)	H20A—C20—H20C	109.5
C10—C9—C14	118.89 (11)	H20B—C20—H20C	109.5
C11—C10—C9	120.90 (12)	C19—C21—H21A	109.5
C11—C10—H10A	119.6	C19—C21—H21B	109.5
C9—C10—H10A	119.6	H21A—C21—H21B	109.5
C10—C11—C12	120.20 (12)	C19—C21—H21C	109.5
C10—C11—H11A	119.9	H21A—C21—H21C	109.5
C12—C11—H11A	119.9	H21B—C21—H21C	109.5
			
C6—C1—C2—C3	−1.50 (19)	N2—C9—C14—C13	177.58 (11)
C1—C2—C3—C4	0.8 (2)	C10—C9—C14—C13	−1.70 (16)
C2—C3—C4—C5	0.58 (18)	N2—C9—C14—C15	−2.51 (16)
C2—C3—C4—N1	−179.23 (12)	C10—C9—C14—C15	178.21 (10)
C8—N1—C4—C5	−3.85 (19)	C17—N3—C15—C16	−0.25 (12)
C8—N1—C4—C3	175.94 (11)	C18—N3—C15—C16	176.10 (11)
C3—C4—C5—C6	−1.29 (17)	C17—N3—C15—C14	−179.22 (12)
N1—C4—C5—C6	178.50 (11)	C18—N3—C15—C14	−2.9 (2)
C2—C1—C6—C5	0.77 (18)	C13—C14—C15—N3	0.0 (2)
C2—C1—C6—C7	−178.19 (11)	C9—C14—C15—N3	−179.85 (11)
C4—C5—C6—C1	0.63 (17)	C13—C14—C15—C16	−178.78 (11)
C4—C5—C6—C7	179.64 (10)	C9—C14—C15—C16	1.32 (16)
C1—C6—C7—O1	164.52 (12)	C17—N4—C16—C15	−0.49 (13)
C5—C6—C7—O1	−14.48 (16)	C17—N4—C16—C8	177.45 (12)
C1—C6—C7—O2	−15.91 (16)	N3—C15—C16—N4	0.46 (13)
C5—C6—C7—O2	165.09 (10)	C14—C15—C16—N4	179.56 (10)
C9—N2—C8—N1	−176.50 (10)	N3—C15—C16—C8	−177.74 (10)
C9—N2—C8—C16	2.20 (16)	C14—C15—C16—C8	1.36 (17)
C4—N1—C8—N2	3.04 (18)	N2—C8—C16—N4	178.96 (11)
C4—N1—C8—C16	−175.68 (11)	N1—C8—C16—N4	−2.32 (18)
C8—N2—C9—C10	−179.91 (10)	N2—C8—C16—C15	−3.25 (16)
C8—N2—C9—C14	0.79 (17)	N1—C8—C16—C15	175.47 (10)
N2—C9—C10—C11	−178.40 (11)	C16—N4—C17—N3	0.33 (13)
C14—C9—C10—C11	0.94 (18)	C15—N3—C17—N4	−0.05 (13)
C9—C10—C11—C12	0.58 (19)	C18—N3—C17—N4	−176.51 (10)
C10—C11—C12—C13	−1.35 (19)	C17—N3—C18—C19	−104.71 (13)
C11—C12—C13—C14	0.55 (18)	C15—N3—C18—C19	79.60 (15)
C12—C13—C14—C9	0.97 (17)	N3—C18—C19—C21	71.13 (13)
C12—C13—C14—C15	−178.92 (11)	N3—C18—C19—C20	−165.62 (11)

### Hydrogen-bond geometry (Å, °)

**Table:**

*D*—H···*A*	*D*—H	H···*A*	*D*···*A*	*D*—H···*A*
N1—H1N1···O1^i^	0.860 (16)	2.114 (17)	2.9436 (14)	161.8 (15)
O2—H1O2···N4^ii^	0.99 (2)	1.71 (2)	2.6926 (13)	172 (2)
C3—H3A···O1^i^	0.93	2.42	3.1998 (15)	142
C5—H5A···N2	0.93	2.21	2.8337 (16)	123
C1—H1A···Cg2^iii^	0.93	2.94	3.3877 (14)	111

Symmetry codes: (i) *x*, −*y*−1/2, *z*+1/2; (ii) *x*, −*y*−1/2, *z*−1/2; (iii) −*x*, *y*−1/2, −*z*+1/2.
